# Identification and characterization of DcUCGalT1, a galactosyltransferase responsible for anthocyanin galactosylation in purple carrot (*Daucus carota* L.) taproots

**DOI:** 10.1038/srep27356

**Published:** 2016-06-06

**Authors:** Zhi-Sheng Xu, Jing Ma, Feng Wang, Hong-Yu Ma, Qiu-Xia Wang, Ai-Sheng Xiong

**Affiliations:** 1 State Key Laboratory of Crop Genetics and Germplasm Enhancement, College of Horticulture, Nanjing Agricultural University, Nanjing, 210095, China; 2 College of Plant Protection, Nanjing Agricultural University, Nanjing, 210095, China

## Abstract

Purple carrots (*Daucus carota* ssp. *sativus* var. *atrorubens* Alef.) accumulate large amounts of cyanidin-based anthocyanins in their taproots. Cyanidin can be glycosylated with galactose, xylose, and glucose in sequence by glycosyltransferases resulting in cyanidin 3-xylosyl (glucosyl) galactosides in purple carrots. The first step in the glycosylation of cyanidin is catalysis by UDP-galactose: cyanidin galactosyltransferase (UCGalT) transferring the galactosyl moiety from UDP-galactose to cyanidin. In the present study, a gene from ‘Deep purple’ carrot, *DcUCGalT1*, was cloned and heterologously expressed in *E. coli* BL21 (DE3). The recombinant DcUCGalT1 galactosylated cyanidin to produce cyanidin-3-*O*-galactoside and showed optimal activity for cyanidin at 30 °C and pH 8.6. It showed lower galactosylation activity for peonidin, pelargonidin, kaempferol and quercetin. It accepted only UDP-galactose as a glycosyl donor when cyanidin was used as an aglycone. The expression level of *DcUCGalT1* was positively correlated with anthocyanin biosynthesis in carrots. The enzyme extractions from ‘Deep purple’ exhibited galactosylation activity for cyanidin, peonidin and pelargonidin, while those from ‘Kuroda’ (a non-purple cultivar) did not.

Numerous publications have proposed that anthocyanins are protective compounds in human health^1^,^2^,^3^,^4^,^5^. These health-promoting effects have made anthocyanin-rich foods increasingly popular. Therefore, much research has focused on exploring the biosynthesis of anthocyanins in plants to improve their dietary content^6^,^7^,^8^,^9^,^10^,^11^. Purple carrots (*Daucus carota* ssp. *sativus* var. *atrorubens* Alef.) contain extremely high levels of anthocyanins, up to 1,750 mg/kg fresh weight in some varieties^12^, which are widely used as a healthy natural food colorant in beverages, candies and ice cream^13^,^14^. Purple carrots are the third largest natural commercial source for anthocyanins^15^. Almost all anthocyanins in purple carrots are based on cyanidin. However, trace amounts of peonidin- and pelargonidin-type aglycones have also been found in purple carrots (Fig. 1)^16^. In purple carrots, the major anthocyanins are 3-xylosyl(glucosyl)galactosides acylated with sinapic, ferulic, hydroxybenzoic, or coumaric acids attached to the glucosyl moiety^17^,^18^.

Glycosyltransferases play an important role in the biosynthesis of anthocyanins because glycosylation usually improves the stability and water solubility of anthocyanidins^19^. Galactosyltransferase has been reported as catalyzing the first step in the glycosylation of cyanidin, where the galactosyl moiety is transferred from UDP-galactose to the 3-*O*-position of cyanidin (Fig. 1)^17^. Glycosyltransferases have selective substrate specificities towards aglycones and sugar donors. Although many glycosyltransferases from various species have shown glycosylation activity for anthocyanins in previous investigations, only those from carrots^20^, peach (*Prunus persica*)^21^, udo (*Aralia cordata* Thunb.)^22^,^23^ and kiwifruit (*Actinidia chinensis*)^24^ have been confirmed to be UDP-galactose:cyanidin galactosyltransferase (UCGalT). The UCGalT purified from carrot showed activity for cyanidin, but the gene which encodes this protein has not yet been confirmed^20^.

In the present study, we cloned a gene of UCGalT from purple carrot (*DcUCGalT1*) and determined its ability to galactosylate cyanidin, peonidin, pelargonidin, kaempferol and quercetin. We also investigated the relationship between the *DcUCGalT1* expression levels and anthocyanin accumulation to understand the importance of the role of *DcUCGalT1* in the biosynthesis of anthocyanins in purple carrot taproots.

## Results

### Nucleotide sequence and deduced amino acid sequence of *DcUCGalT1*

DcUCGalT1 was identified by amino acid sequence similarity to *A. cordata* anthocyanin 3-*O*-glactosytransferase (Accession No. AB103471) with the BLAST tool in the CarrotDB (http://apiaceae.njau.edu.cn/carrotdb/index.php). The nucleotide sequence of *DcUCGalT1* has been submitted to the GenBank with accession number KP319022. *DcUCGalT1* contained a 1,356 bp open reading frame encoding a polypeptide of 452 amino acids. The calculated molecular mass of native DcUCGalT1 was 49.37 kDa.

**Figure 2:**
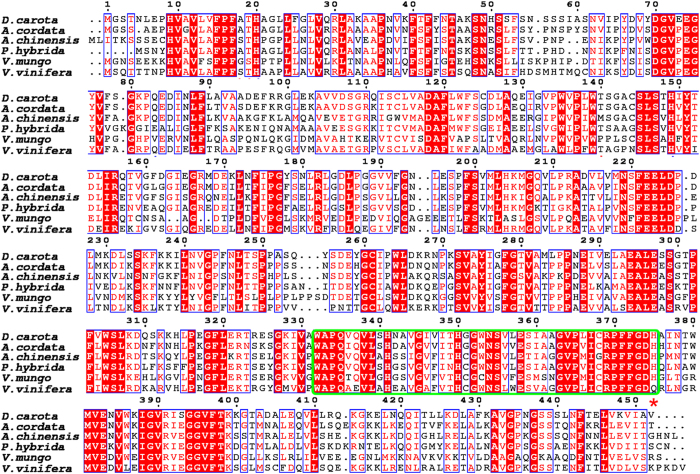
Alignment of deduced amino acid sequences of DcUCGalT1 with anthocyanidin galactosyltransferases from *Aralia cordata* (Accession No. AB103471) and *Actinidia chinensis* (Accession No. GU079683), flavonoid galactosyltransferases from *Petunia hybrida* (Accession No. AF165148) and *Vigna mungo* (Accession No. AB009370), and anthocyanidin glucosyltransferase from *Vitis vinifera* (Accession No. AF000372). The UGT signature PSPG motifs are enclosed in the green box. The last residues of PSPG motifs are indicated with a red asterisk (*) below the alignment.

The deduced amino acid sequence of DcUCGalT1 was compared with that of two anthocyanidin galactosyltransferases from *Aralia cordata* and *Actinidia chinensis*, two flavonoid galactosyltransferases from *Petunia hybrida* and *Vigna mungo* as well as anthocyanidin glucosyltransferase from *Vitis vinifera* (VvGT1) using the ESPript3.0 website^25^. Among these UGTs, the amino acid sequence of DcUCGalT1 showed the highest identity with the UCGalT from *A. cordata* (Fig. 2). Like other UGTs, the UDP-sugar binding PSPG (Putative Secondary Plant Glycosyltransferase) motif also exists in the C-terminal domain of DcUCGalT1 (Residues Trp332-His375). Histidine is specifically conserved as the last amino acid residue of the PSPG motif in galactosyltransferases^23^. However, the last amino acid residue of the PSPG motif in glucosyltransferase, such as VvGT1, is always a glutamine (Fig. 2). Like the other four anthocyanindin galactosyltransferases, the last amino acid of the deduced PSPG motif in the DcUCGalT1 is also histidine. Thus DcUCGalT1 is deduced to be a cyanidin-3-*O*-galactosyltransferase.

### Expression of DcUCGalT1 in *E. coli* and purification of recombinant DcUCGalT1

**Figure 3:**
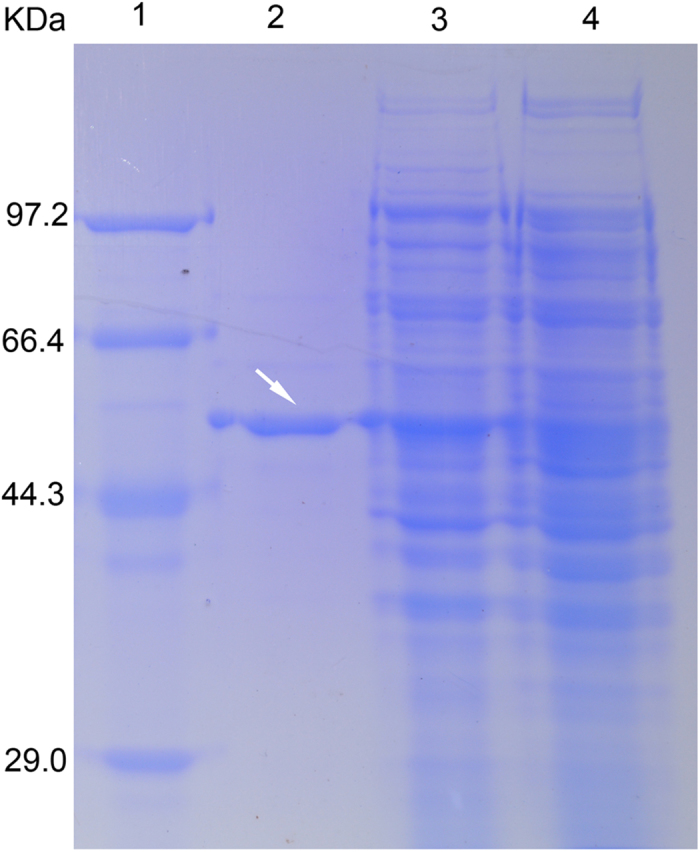
Coomassie stained SDS-PAGE of rDcUCGalT1 from expression in *E. coli*. Lane 1, protein size standards; Lane 2, purified rDcUCGalT1 (white arrow); Lane 3, total soluble protein from BL21(DE3) cells containing the DcUCGalT1 plasmid following induction with IPTG; Lane 4, total soluble protein from BL21(DE3) cells containing the DcUCGalT1 plasmid without induction.

*DcUCGalT1* was cloned into the pET30 vector, which resulted in addition of His-tag, thrombin, S-tag, and enterokinase sequences to the N-terminus of the protein. The exact sequences of recombinant DcUCGalT1 (rDcUCGalT1) actually being expressed in *E. coli* are shown in Supplementary Fig. S1. The rDcUCGalT1 was successfully expressed in *E. coli* BL21(DE3) in the presence of 1.0 mM IPTG for over 12 h at 18 °C. The molecular mass of rDcUCGalT1 was calculated as 54.80 kDa, while SDS-PAGE analysis showed a prominent band of the purified enzyme at about 55 kDa (Fig. 3, white arrow).

### UFGT activity of rDcUCGalT1

**Figure 4:**
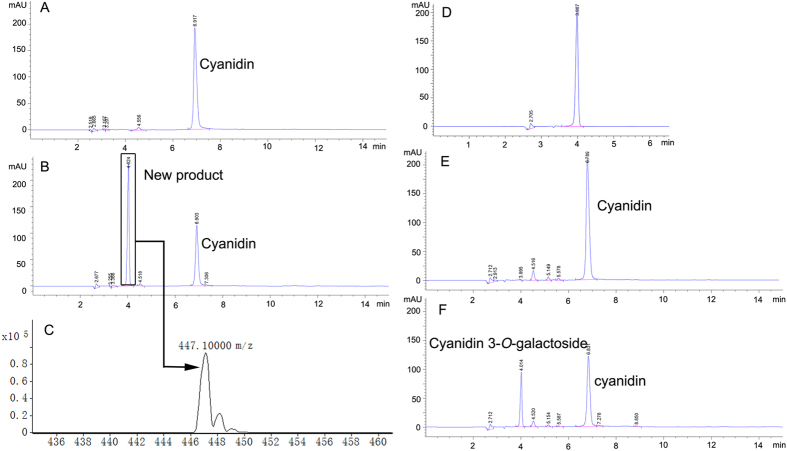
HPLC and LC-MS analyses of the enzymatic reaction products (Diode Array Detector (DAD) detection at 530 nm shown). (**A,B**) HPLC chromatogram showing peaks for products after control enzyme from *E.coli* (**A**) or rDcUCGalT1 (**B**) incubated with cyanidin and UDP-galactose. (**C**) LC–MS analysis of new product after rDcUCGalT1 enzymatic reaction. (**D**) HPLC chromatogram showing peaks for cyanidin-3-*O*-galactoside standard. (**E,F**) HPLC chromatogram showing peaks for products after the crude enzyme from ‘Kuroda’ (**E**) and ‘Deep purple’ (**F**) incubated with cyanidin and UDP-galactose.

The glycosylation ability of rDcUCGalT1 towards cyanidin and four other flavonoid substrates was determined. The rDcUCGalT1 glycosylated cyanidin to form a new product by using UDP-galactose as the glycosyl donor (Fig. 4A,B). The new product showed the same retention time as cyanidin 3-*O*-galactoside standard (Fig. 4D) and showed an ion at m/z 447.1 in the LC-MS spectral data (Fig. 4C), thus confirming that the new product was cyanidin 3-*O*-galactoside.

The rDcUCGalT1 also conjugated peonidin, pelargonidin, kaempferol and quercetin with UDP-galactose as the glycosyl donor (See Supplementary Fig. S2). However, rDcUCGalT1 showed no glycosylation activity towards cyanidin with UDP-glucose or UDP-xylose as glycosyl donors (Table 1). The activity of the enzyme for cyanidin was the highest among all the acceptor substrates tested (Table 1). In addition, rDcUCGalT1 accepted only UDP-galactose as a glycosyl donor among the UDP sugars tested in the present study.

### Optimal temperature and pH for rDcUCGalT1 activity towards cyanidin

**Figure 5:**
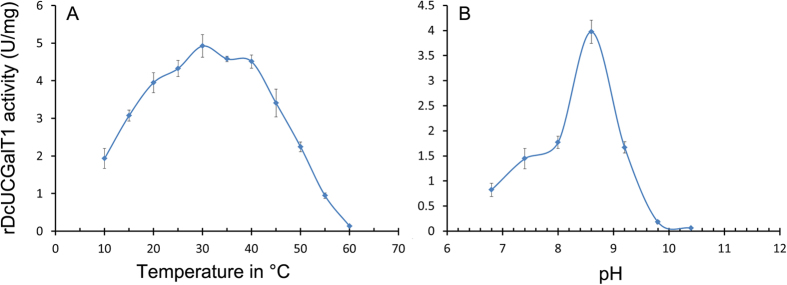
rDcUCGalT1 activities at different temperature and pH. Effects of temperature (**A**) and pH (**B**) on rDcUCGalT1 activity with cyanidin and UDP-galactose as substrates. The values are the mean of three separate experiments with error bars showing ± SD.

rDcUCGalT1 activity towards cyanidin was determined at temperatures from 10–60 °C with maximum activity being observed at 30 °C (Fig. 5A). rDcUCGalT1 activity was barely detectable when the temperature reached 60 °C. The enzyme activity was also tested over the pH range of 6.8–10.4. rDcUCGalT1 showed maximum activity at pH 8.6 in the pH range tested (Fig. 5B). With an increase or decrease from pH 8.6, rDcUCGalT1 activity gradually decreased, becoming barely detectable when the pH reached 10.4.

### Kinetic Analyses of rDcUCGalT1

Purified rDcUCGalT1was used to determine the apparent *K*_m_ and *V*_max_ values of cyanidin, peonidin and pelargonidin (Table 2), as the three anthocyanins are endogenous in purple carrots. Using a saturating concentration of UDP-galactose, the apparent *K*_m_ values for cyanidin, peonidin, and pelargonidin were calculated as 65.57 ± 5.14, 140.34 ± 14.63 and 40.97 ± 3.78 μM, respectively and the apparent *V*_max_ values as 11.17 ± 0.30, 7.23 ± 0.62 and 1.32 ± 0.10 μmol min^−1^ mg^−1^, respectively. Using a fixed concentration of cyanidin, peonidin, and pelargonidin, the apparent *K*_m_ values of UDP-galactose were determined as 398.394 ± 16.83, 1482.34 ± 134.54 and 749.58 ± 61.29 μM, with apparent *V*_max_ values of 6.28 ± 0.18, 3.05 ± 0.37 and 0.75 ± 1.9 × 10^−3^μmol min^−1^mg^−1^, respectively.

### Glycosylation of anthocyanins with crude enzyme from taproots of carrots

The crude enzyme was extracted from the taproots of ‘Deep purple’ and ‘Kuroda’ carrot cultivars (See Supplementary Fig. S3). The glycosylation activity of the crude enzyme was determined using reverse phase HPLC. The crude enzyme from ‘Kuroda’ did not conjugate cyanidin with UDP-galactose as the glycosyl donor (Fig. 4E). However, that from ‘Deep purple’ glycosylated cyanidin with the same donor substrate (Fig. 4F). The crude enzyme from ‘Deep purple’ also showed glycosylation activity towards peonidin and pelargonidin but the crude enzyme from ‘Kuroda’ did not (See Supplementary Fig. S4).

### Expression profiles of *DcUCGalT1* in the taproots of carrots

**Figure 6:**
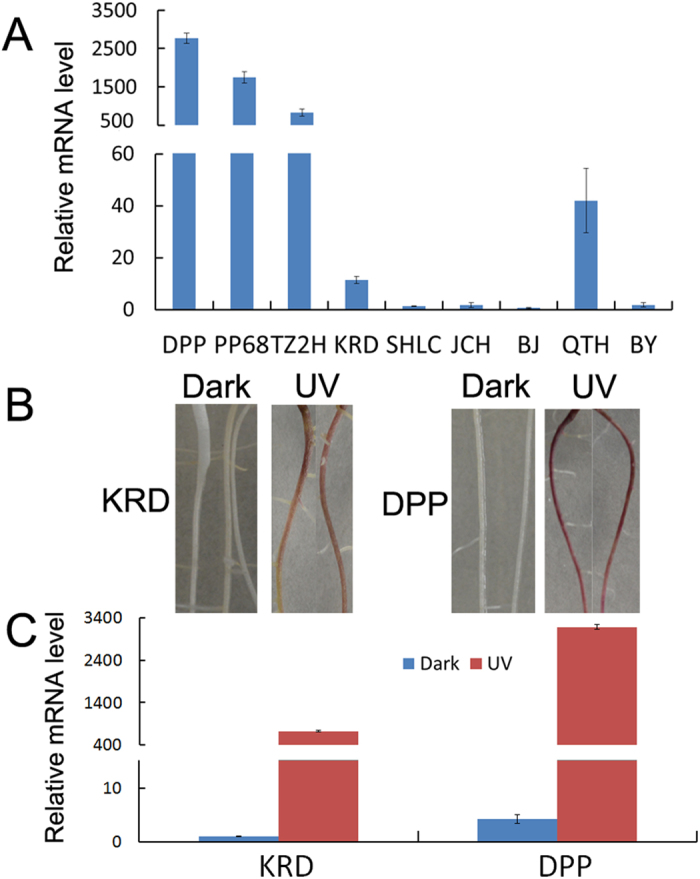
Expression of *DcUCGalT1* in carrots. (**A**) Expression of *DcUCGalT1* in 60-day-old taproots of 3 purple carrot cultivars and 6 non-purple carrot cultivars. (**B**) The roots of seedlings of ‘Kuroda’ and ‘Deep purple’ grown in the dark or under continuous irradiation with UV light. (**C**) Expression of *DcUCGalT1* in ‘Kuroda’ and ‘Deep purple’ after UV irradiation or in the dark. Data are the means of three biological replicates with error bars showing ± SD. Cultivar abbreviations: DPP, Deep purple; PP68, Purple 68; TZ2H, Tianzi2hao; KRD, Kuroda; SHLC, Sanhongliucun; JCH, Junchuanhong; BJ, Bejo1719; QTH, Qitouhuang; BY, Baiyu.

The three purple carrot cultivars (Deep purple, Purple68 and Tianzi2hao) accumulate anthocyanins in the 60-day-old taproots but the six non-purple carrot cultivars (Kuroda, Sanhongliucun, Junchuanhong, Bejo1719, Qitouhuang and Baiyu) do not when grown in the soil^26^. The expression profiles of *DcUCGalT1* were analyzed in 60-day-old taproots of the three purple carrot cultivars and six non-purple carrot cultivars (Fig. 6A). *DcUCGalT1* showed high transcript abundance in the purple taproots of ‘Deep purple’, ‘Purple 68’, and ‘Tianzi2hao’, very low RNA levels in ‘Qitouhuang’and ‘Kuroda’, trace RNA levels in ‘Sanhongliucun’, ‘Junchuanhong’, ‘Bejo1719’, and ‘Baiyu’.

Ultraviolet (UV) light irradiation significantly enchanced carrot anthocyanin accumulation in previous reports^17^,^27^. The ‘Kuroda’ carrot taproots don’t accumulate anthocyanin at any time when grown in soil. For ‘Deep purple’ carrot grown in soil, the taproots don’t accumulate any anthocyanin in the first two weeks, although they accumulate rich anthocyanins when they are mature. However roots of both cultivars did accumulate anthocyanins when exposed to UV light. In this study, the Petri dishes planted with carrots seeds were placed vertically and the carrot seedlings were grown along the surface of the solid medium so that UV-light could stimulate the entire roots of carrots. The roots of 12-day-old seedlings of ‘Kuroda’ and ‘Deep purple’ both turned purple after UV-light irradiation, suggesting that UV-light induced anthocyanin accumulation in carrot roots (Fig. 6B). With UV-light irradiation, the roots of 12-day-old seedlings of ‘Deep purple’ showed deeper purple color than that of ‘Kuroda’. However, the roots of 12-day-old seedlings of ‘Kuroda’ and ‘Deep purple’ were white when grown in the dark, which suggests that anthocyanins hadn’t accumulated in the roots (Fig. 6B). The expression profiles of *DcUCGalT1* in the roots of 12-day-old seedlings of ‘Kuroda’ and ‘Deep purple’ were also determined. The abundance of *DcUCGalT1* transcript was much higher in the roots of 12-day-old seedlings of ‘Kuroda’ and ‘Deep purple’ with UV irradiation than that of ‘Kuroda’ and ‘Deep purple’ grown in the dark (Fig. 6C). With UV-light irradiation, the expression levels of *DcUCGalT1* in the roots of 12-day-old seedlings of ‘Deep purple’ were higher than that of ‘Kuroda’.

## Discussion

Purple carrot cultivars accumulate large amounts of anthocyanins in their taproots. The predominant anthocyanins in purple carrot taproots are derived from cyanidin^18^. UCGalT was thought to catalyze the first step in the glycosylation of cyanidin, which transferred the galactosyl moiety from UDP-galactose to the 3-*O*-position of cyanidin^17^. In the present study, a cDNA clone, namely *DcUCGalT1*, was obtained and sequenced from a purple carrot cultivar (Deep purple). Of those analyzed, the identity of the deduced amino acid sequence of DcUCGalT1 was closest to that of UCGalT from *A. cordata*, which may be because *D. carota* and *A. cordata* both belong to the Apiales order. The molecular mass of glycosyltransferases generally ranges from 45 to 60 kDa^23^. That of DcUCGalT1 was calculated to be 49.37 kDa, a little lower than a native UCGalT (52 kDa) from *D. carota* which was determined using a Sephadex G-75 column^20^.

The rDcUCGalT1 activity was monitored and confirmed by measuring the formation of cyanidin-3-*O*-galactoside using HPLC and LC-MS analyses. These data suggest that rDcUCGalT1 transferred a galactose from UDP-galactose to the 3-OH of cyanidin. The optimal galactosyltransferase activity of rDcUCGalT1 for cyanidin was obtained at 30 °C and at pH 8.6, higher than the optimum temperature of 23 °C and pH 6.8 previously reported for a native UCGalT from *D. carota*^20^, which may be caused by the addition of a His-tag, thrombin, S-tag, and enterokinase sequences to the N-terminus of the rDcUCGalT1protein. The optimal pH for rDcUCGalT1 activity was slightly alkaline, similar to other UFGTs^28^,^29^.

Peonidin- and pelargonidin-derived anthocyanins have previously been found in purple carrots^18^. In the present study, rDcUCGalT1 also glycosylated peonidin and pelargonidin. The activity of rDcUCGalT1 for cyanidin was highest among all three endogenous anthocyanins in purple carrots, which may partly explain why purple carrots accumulated much more cyanidin-based anthocyanins than those based on peonidin and pelargonidin. In addition, rDcUCGalT1 accepted only UDP-galactose as a glycosyl donor in the present study.

It has been reported that *chalcone synthase 1*, *chalcone–flavonone isomerase 1*, *flavanone 3-hydroxylase 1*, *flavonoid 3′-monooxygenase 1*, *dihydroflavonol 4-reductase 1*, and *leucoanthocyanidin dioxygenase 1*/*leucoanthocyanidin dioxygenase 2* were highly expressed in purple carrot taproots but not or scarcely expressed in non-purple carrot taproots at the 60-day-old stage^26^. The expression of these genes appeared to be the determining steps in the production of anthocyanins in purple carrots^26^. In the present study, using qRT-PCR, we have described the expression patterns of *DcUCGalT1* in the taproots of purple and non-purple carrot cultivars at 60 days. We have found that *DcUCGalT1* was highly expressed in the taproots of all three 60-day-old purple carrot cultivars. However, the *DcUCGalT1* transcript was not or scarcely detectable in the 60-day-old taproots of the six non-purple carrot cultivars, showing a similar pattern to that described in grapevines^30^ and kiwifruit^24^. Anthocyanin content increased in 12-day-old roots of ‘Deep purple’ and ‘Kuroda’ after UV irradiation. Similar to UCGalT from *D. carota* reported previously^20^, the expression levels of *DcUCGalT1* also increased both in ‘Deep purple ’ and ‘Kuroda’ after UV irradiation. These results suggest that *DcUCG*alT1 was positively correlated with anthocyanin biosynthesis in carrot taproots. The crude enzyme extractions from ‘Deep purple’ showed galactosylation activity predominantly for cyanidin, as well as peonidin and pelargonidin, while those from ‘Kuroda’ did not. In purple carrots, galactosyltransferases catalyze the first step in the glycosylation of many different kinds of the anthocyandins^2^. The glycosylation step is critical for stability and water solubility of anthocyanidins, producing the first stable coloured compounds of the anthocyanin biosynthetic pathway^24^,^31^. Loss of activity of UDP-flavonoid 3-*O*-galactosyltransferase leads to no accumulation of anthocyanin in kiwifruit^24^.

In some other plant species, transcription factors, especially MYBs, were reported to control the transcription of structural genes for anthocyanin biosynthesis, including *UFGTs*^32^,^33^,^34^. That *DcUCG*alT1 was not or was scarcely expressed in non-purple carrots is possibly caused by the lack of activation by transcription factors. In future work, we will focus on indentification of transcription factors which control expression of *DcUCG*alT1.

## Methods

### RNA extraction and cDNA synthesis

The total RNA was extracted from the carrots using an RNA Simple Total RNA Kit (Tiangen, Beijing, China) following the manufacturer’s instructions. cDNA was synthesized as described previously^26^ and was diluted 20-fold for gene cloning and qRT-PCR analysis.

### Gene searching, cloning, and sequencing

The nucleotide sequence of *DcUCGalT1* gene was found using CarrotDB: a genomic and transcriptomic database for carrot built by our group (http://apiaceae.njau.edu.cn/carrotdb/index.php)^35^. The gene was cloned from ‘Deep purple’ carrots by PCR using a forward primer: 5′-ATGGGGAGTACAAATCTGGAAC-3′ and a reverse primer: 5′-TCAGACAGCAATCACTTTTACTAGC-3′ and verified by sequencing with an Applied Biosystems 3730 DNA Analyzer (Genscript, Nanjing, China).

### DcUCGalT1 cDNA expression in Escherichia coli

By using the ClonExpress II One Step Cloning Kit (Vazyme Biotech Co. Ltd., Nanjing, China), the coding sequence of *DcUCGalT1* was subcloned into a pET-30a(+) expression vector (Novagen, Darmstadt, Germany) between the *Bam*HI/*Sac*I sites using a forward primer: 5′-GCCATGGCTGATATCGGATCCATGGGGAGTACAAATCTGGAAC-3′ and a reverse primer: 5′-ACGGAGCTCGAATTCGGATCCTCAGACAGCAATCACTTTTACTAGC-3′, followed by transformation into *E. coli* DH5α. The authenticity of the resulting clones was confirmed by PCR analysis and sequencing. The recombinant plasmid pET-30a (+)-DcUCGalT1 was extracted from the positive clones and then transformed into *E. coli* BL21(DE3) cells (TransGen, Beijing, China). The BL21(DE3) cells containing recombinant plasmid were grown in 50 mL LB medium containing 50 mg L^−1^ kanamyacin at 37 °C for 4-5 h while being shaken at 230 rpm until an OD_600_ of 0.4–0.6 was attained. The recombinant protein was induced by adding isopropyl β-D-1-thiogalactopyranoside (IPTG) to a final concentration of 1.0 mM to the culture and adjusting the temperature to 18 °C. The culture was incubated for at least 12 h.

### Recombinant protein purification

Cells were harvested by centrifugation, re-suspended in 2 mL lysis buffer (pH = 7.5) containing 50 mM NaH_2_PO_4_, 300 mM NaCl, 10% glycerol, 10 mM β-mercaptoethanol and 10 mM imidazole. The cell suspension was then sonicated for 20 min on ice. The cell debris in the sonicated mixture was removed by centrifugation at 12,000 × *g* for 10 min at 4 °C then filtered using 0.22-μm microfiltration membranes. The soluble mixture was passed through a column containing Ni-NTA-agarose resin (1.5 mL bed volume) (Qiagen, Hilden, Germany). The resin was washed with 1 mL wash buffer (50 mM NaH_2_PO_4_, 300 mM NaCl, 10% glycerol, 10 mM β-mercaptoethanol and 50 mM imidazole, pH = 7.5) six times. The 6 × His-tagged recombinant protein was then eluted from the resin with 2 mL elution buffer (50 mM NaH_2_PO_4_, 300 mM NaCl, 10% glycerol, 10 mM β-mercaptoethanol and 250 mM imidazole, pH = 7.5). The recombinant protein fraction was desalted against a buffer (50 mM NaH_2_PO_4_-Na_2_HPO_4_, pH = 7.5, 0.1 mM EDTA, 1 mM DTT) using a HiTrap desalting column (GE Healthcare Life Sciences, Beijing, China) according to the manufacturer’s instructions. Then a protease inhibitor cocktail (Product Number: P8849; Sigma, St. Louis, USA) was added to the purified protein fraction (10 μL/1 mL). The purified protein concentration in the fractions was quantified using a Bradford assay kit with bovine serum albumin (BSA) as standard and analyzed using 12% SDS-PAGE with Coomassie staining.

### rDcUCGalT1 activity assay

The rDcUCGalT1 activity was assayed in 50 mM NaH_2_PO_4_-Na_2_HPO_4_ (pH = 7.5), 1 mM UDP-galactose,-glucose, or -xylose, 0.2 mM acceptor substrates, cyanidin, peonidin, pelargonidin, kaempferol and quercetin (Sigma), and about 0.1–0.5 μg of purified rDcUCGalT1 in a final volume of 50 μL. About 0.1–0.5 μg of the total soluble protein from BL21(DE3) cells containing the DcUCGalT1 plasmid without induction was used as the control. The reaction was initiated by adding various flavonoids after the mixture was incubated at 30 °C for 3 min. After further incubation at 30 °C for 5 min, the reaction was terminated by adding 10 μL 12 M HCl, vortexed for 60 s then centrifuged at 12,000 × *g* for 10 min to precipitate proteins. Twenty μL of the supernatant was analyzed by HPLC to detect and quantify the glycosylated products. A 1200 series HPLC (Agilent Technologies, Palo Alto, USA) with an Inertsil OPD -SP column (C18, 4.6 mm × 250 mm, particle size 5 μm, GL Sciences, Inc., USA) was used for separating the reactants and the products. Cyanidin, peonidin and pelargonidin were separated from their glycosides using a linear gradient from 20% solvent A (acetonitrile) and 80% solvent B (0.5% acetic acid in water) to 40% of A and 60% of B in 10 min at 1.0 mL/min, using diode array detection at 530 nm. The conditions used for separating kaempferol and quercetin from their glycosides were as follows: a linear gradient of 20% solvent A and 80% B to 90% of A and 10% of B in 10 min at 1.0 mL/min, 360 nm.

Liquid chromatography-mass spectrometry (LC-MS) was used to determine the glycoside product of cyanidin using 20 μL of the reaction mixture. The conditions used for elution were as follows: a linear gradient of 20% solvent A and 80% solvent B to 40% of A and 60% of B in 20 min at 0.5 mL/min, 530 nm. The peak corresponding to the product was subjected to negative ion mass spectrometry analysis (MS) with the same other conditions as described previously^36^.

### Effect of pH and temperature on rUCGalT1 activity

The optimum pH for enzyme activity was determined in 50 mM NaH_2_PO_4_-Na_2_HPO_4_ (pH = 6.8–8.0) and 50 mM glycine-NaOH (pH = 8.6–10.4) at 40 °C for 5 min. The effect of temperature (10–60 °C) on enzyme activity was determined at pH 8.6 in 50 mM glycine-NaOH for 3 min.

### Kinetic analyses of rUCGalT1 towards anthocyanidins

The initial velocity versus substrate concentration curves for the rUCGalT1 reaction were determined using a fixed anthocyanidin (cyanidin, peonidin, and pelargonidin) concentration of 0.2 mM and varying the concentration of UDP-galactose from 0.25–4 mM, or using a fixed UDP-galactose concentration of 2 mM and varying the concentration of anthocyanidins (0.025–0.2 mM for cyanidin, 0.02–0.4 mM for peonidin and 0.025–0.2 mM for pelargonidin). The reactions took place in 50 mM glycine-NaOH (pH = 8.6) at 30 °C. The apparent *K*_m_ and *V*_max_ values were estimated using Hanes-Woolf plots.

### Extraction and activity assay of enzyme from the taproots of ‘Deep purple’ and ‘Kuroda’

The 60-day old taproots of ‘Deep purple’ and ‘Kuroda’ were grown under the same conditions as previously described^26^ and were frozen in liquid nitrogen then ground to a fine powder. The powdered tissue (about 0.2 g) was extracted in 1 mL of extraction buffer (10 mM NaH_2_PO_4_-Na_2_HPO_4_, pH 7.5, 1 mM DTT and 0.1 mM EDTA) by vortexing briefly for mixing. The cell debris in the mixture was removed by centrifugation at 12,000 × g for 10 min at 4 °C then filtration with 0.22-μm microfiltration membranes. The supernatant was then injected into a HiTrap desalting column (GE) against the extraction buffer to remove the anthocyanins. A protease inhibitor cocktail (10 μL /mL) (Product Number: P9599; Sigma) was then added to the extracts that were used as the source of enzymes. All steps in enzyme extraction were carried out at 4 °C with the protein concentration being quantified using a Bradford assay kit.

The UCGalT activity was assayed in a mixture (50 μL) containing 8 μg of enzyme extract, 50 mM glycine-NaOH (pH = 8.6), 1 mM UDP-galactose, 0.2 mM acceptor substrates (cyanidin, peonidin and pelargonidin) which was incubated at 30 °C for 30 min. Five μL of 12 M HCl was added to the reaction mixture then centrifuged to precipitate proteins. A total of 20 μL of the supernatant was analyzed using HPLC using the method outlined above.

### Detection of the expression patterns of *DcUCGalT1* in taproots of purple and non-purple carrots

Three purple carrot cultivars (Deep purple, Purple 68 and Tianzi2hao) and six non-purple carrot cultivars (Kuroda, Sanhongliucun, Junchuanhong, Bejo1719, Qitouhuang and Baiyu) were grown in the soil under the same conditions as previously described^26^. The total RNA was extracted from the 60-day-old carrot taproots using the method as previously described^26^. cDNA was synthesized and was diluted 20-fold for detecting gene expression levels by qRT-PCR analysis using the method as previously described^26^.

### Detecting the effect of UV-light irradiation on the *DcUCGalT1* expression patterns in ‘Deep purple’ and ‘Kuroda’

The seeds of ‘Deep purple’ and ‘Kuroda’ were sterilized as follows: soaked in tap water for 12 h, immersion in 70% (v /v) ethanol for 1 min, soaking in sodium hypochlorite (1.0% available chlorine) containing a drop of Tween™ 20 for 45 min, rinsing with sterile tap water three times, and transferring onto filter papers to remove excess water. After sterilization, seeds were placed onto solid Murashige and Skoog medium^37^ in 90 × 15 -mm Petri dishes, and then were placed vertically at 25 °C in the dark or with continuous UV-containing white light (315–420 nm)^17^ irradiation for 12 days. The total RNA was extracted from the 12-day-old roots using the method as previously described^26^. cDNA was synthesized and was diluted 20-fold for detecting gene expression levels by qRT-PCR analysis using the method as previously described^26^.

### Quantitative real-time polymerase chain reaction (qRT-PCR) expression analysis

The forward primer 5′-ATTCGAGGAACTAGACCCTGACC-3′ and reverse primer 5′-GCCACAGACTTAGGATTACGCTTG-3′ were designed using Primer 5 for qRT-PCR expression analysis. qRT-PCR was performed as described previously^26^. The *DcActin1* gene was used as an internal standard for normalization^26^. The experiments were conducted in triplicate using three biological RNA samples for each carrot cultivar. The relative gene expression was calculated with the 2^−ΔΔCT^ method^38^. For comparing the expression patterns of *DcUCGalT1* among purple and non-purple carrots, the ΔΔC_T_ was calculated by subtracting ΔC_T_ of ‘Sanhongliucun’ from ΔC_T_ of all the carrot cultivars. For detecting the effect of UV-light irradiation on the *DcUCGalT1* expression patterns in ‘Deep purple’ and ‘Kuroda’, the ΔΔC_T_ was calculated by subtracting ΔC_T_ of ‘Kuroda’ under dark from ΔC_T_ of all the samples.

## Additional Information

**How to cite this article**: Xu, Z.-S. *et al.* Identification and characterization of DcUCGalT1, a galactosyltransferase responsible for anthocyanin galactosylation in purple carrot (*Daucus carota* L.) taproots. *Sci. Rep.* **6**, 27356; doi: 10.1038/srep27356 (2016).

## Supplementary Material

Supplementary Information

## Figures and Tables

**Figure 1:**
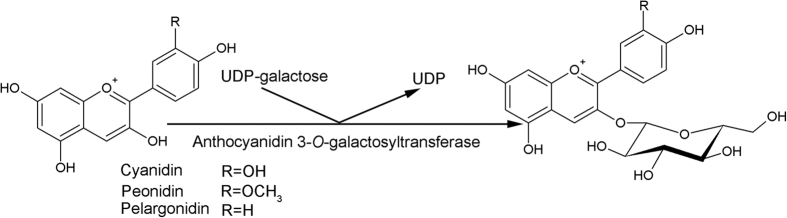
Structures of anthocyanidins and their galactosides found in purple carrot. UCGalT catalyzes the transfer of galactose from UDP-galactose to the 3-OH of anthocyanidins.

**Table 1:** Relative rDcUCGalT1 activity with different substrates.

Substrate	Relative activity (%)
Cyanidin + UDP-galactose	100
Peonidin + UDP-galactose	31.30 ± 2.44
Quercetin + UDP-galactose	22.74 ± 0.54
Kaempferol + UDP-galactose	20.08 ± 0.28
Pelargonidin + UDP-galactose	10.12 ± 0.86
Cyanidin + UDP-glucose	0
Cyanidin + UDP-xylose	0

The activity of rDcUCGalT1 using cyanidin and UDP-galactose as substrates was set as 100. The values represent the mean of three separate experiments ± SD.

**Table 2:** Kinetic parameters of rDcUCGalT1.

Substrate fixed	Substrate varied	*K*_m_(μM)	*V*_max_ (μmol min^−1^ mg^−1^)
UDP-galactose	Cyanidin	65.57 ± 5.14	11.17 ± 0.30
UDP-galactose	Peonidin	140.34 ± 14.63	7.23 ± 0.62
UDP-galactose	Pelargonidin	40.97 ± 3.78	1.32 ± 0.10
Cyanidin	UDP-galactose	398.394 ± 16.83	6.28 ± 0.18
Peonidin	UDP-galactose	1482.34 ± 134.54	3.05 ± 0.37
Pelargonidin	UDP-galactose	749.58 ± 61.29	0.75 ± 1.9 × 10^−3^

The values represent the mean of three separate experiments ± SD.
