# Supplementary material for: Identification and characterization of DcUCGalT1, a galactosyltransferase responsible for anthocyanin galactosylation in purple carrot (Daucus carota L.) taproots
Source: Sci Rep. 2016 Jun 6;6:27356. doi: 10.1038/srep27356 (PMC4893604; doi:10.1038/srep27356)

**Identification and characterization of DcUCGalT1, a galactosyltransferase responsible for anthocyanins galactosylation in purple carrot (*Daucus carota* L.) taproots**

Zhi-Sheng Xu^1^, Jing Ma^1^, Feng Wang^1^, Hong-Yu Ma^2^, Qiu-xia Wang^2^, Ai-Sheng Xiong^1^*

^1^ *State Key Laboratory of Crop Genetics and Germplasm Enhancement, College of Horticulture, Nanjing Agricultural University, Nanjing, 210095, China*

^2^ *College of Plant Protection, Nanjing Agricultural University, Nanjing, 210095, China*

*Please address all correspondence to: A.S. Xiong (xiongaisheng@njau.edu.cn)

-----------------

Dr. Ai-Sheng Xiong

Professor

State Key Laboratory of Crop Genetics and Germplasm Enhancement,

College of Horticulture,

Nanjing Agricultural University,

Nanjing, 210095, China

Tel: 86 25 84396790

Fax: 86 25 84396790

Email: xiongaisheng@njau.edu.cn

***Running title:*** *An anthocyanins galactosyltransferase in purple carrot*

**Supplementary Information**

**Figure S1.** The deduced amino acid sequences of native DcUCGalT1 (nDcUCGalT1) and recombinant DcUCGalT1 (rDcUCGalT1). The calculated molecular mass of nDcUCGalT1 and rDcUCGalT1 were 49.37 kDa and 54.80 kDa, respectively.


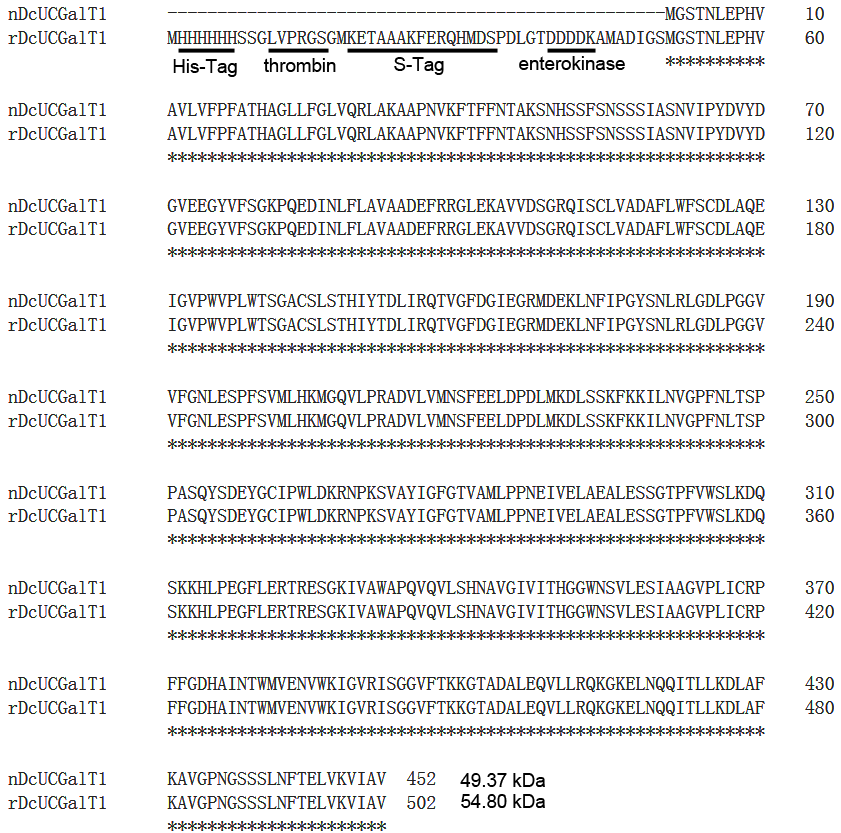


**Figure S2.** Functional analysis of the rDcUCGalT1 enzyme. HPLC chromatogram showing peaks for the products of the following reactions (Detection at 530 nm shown for peonidin and pelargonidin; detection at 360 nm shown for kaempferol and quercetin): (A) UDP-galactose + peonidin + empty vector; (B) rDcUCGalT1; (C) UDP-galactose + pelargonidin + empty vector; (D) rDcUCGalT1; (E) UDP-galactose + kaempferol + empty vector; (F) rDcUCGalT1; (G) UDP-galactose + quercetin + empty vector; (H) rDcUCGalT1.


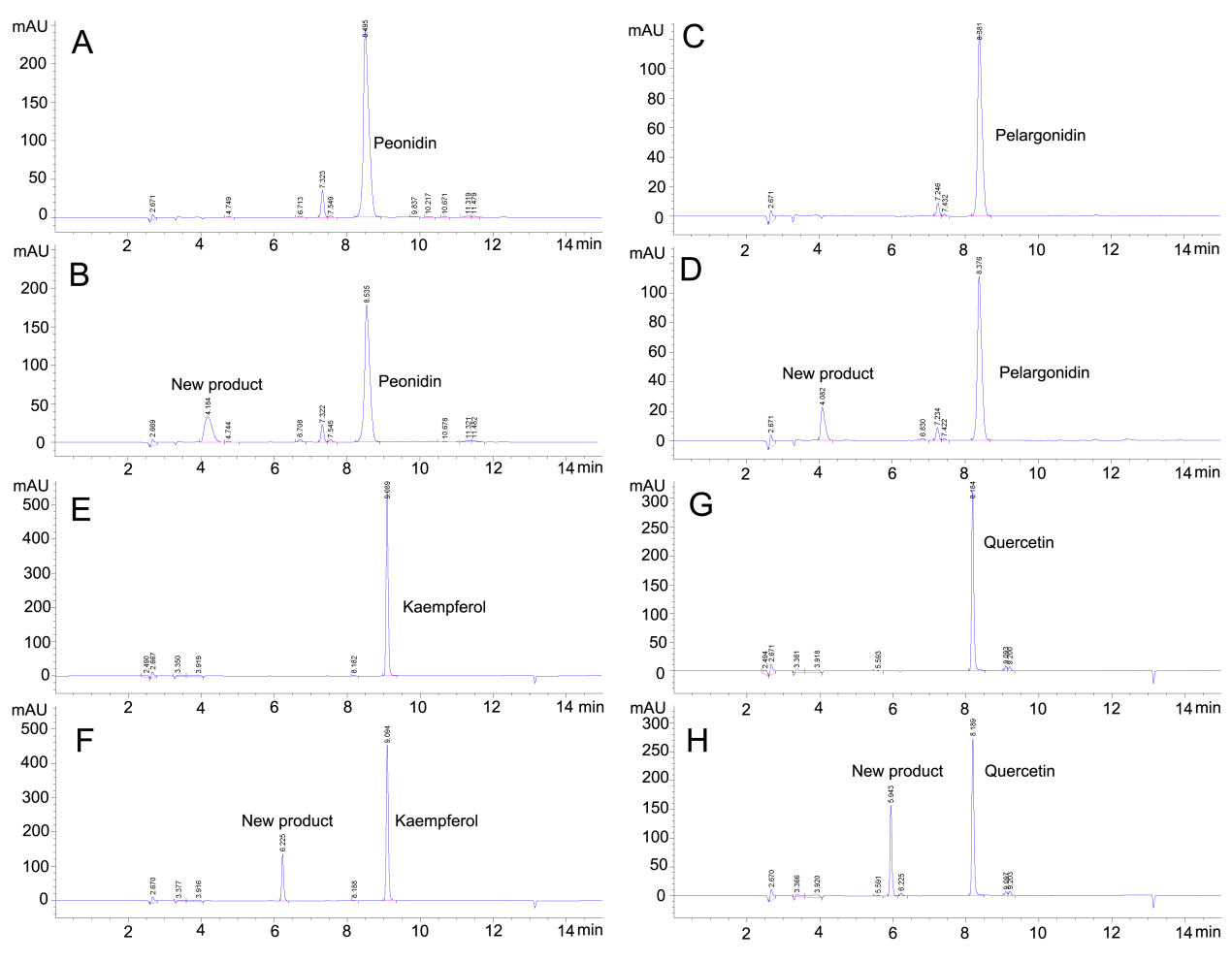


**Figure S3.** The protocol of crude enzyme extraction from taproot of ‘Deep purple’ and ‘Kuroda’. The taproot of (A) ‘Deep purple’ and (B) ‘Kuroda’ used for crude enzyme extraction; (C) powdered taproot of ‘Deep purple’ (a) and ‘Kuroda’ (b) in extraction buffer; (D) The supernatant of extraction after centrifugation; (E) The anthocyanins from ‘Deep purple’ were retained in HiTrap Desalting column (red arrow); (F) The crude enzyme extraction from the taproot of ‘Deep purple’ and ‘Kuroda’.

**
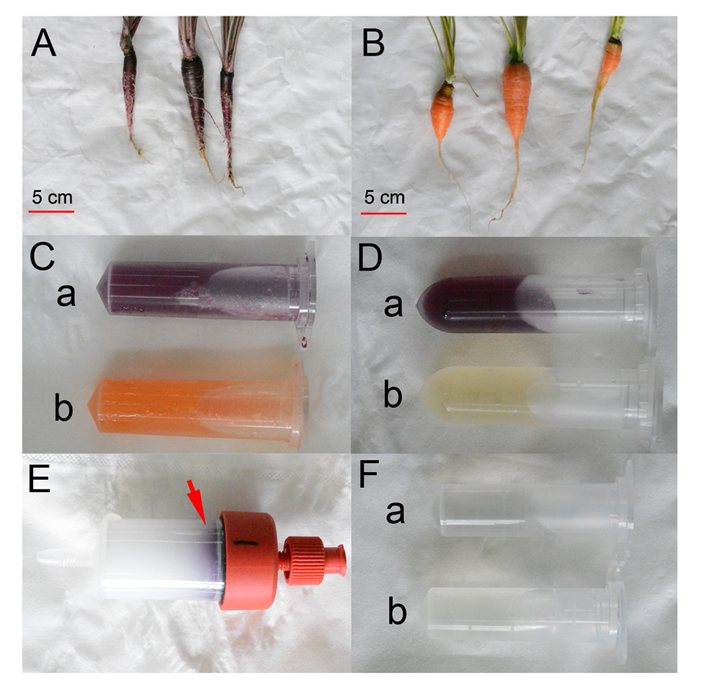
**

**Figure S4.** Functional analysis of the crude enzyme from the taproot of carrot. HPLC chromatogram showing peaks for the products of the following reactions (Detection at 530 nm): the peonidin and UDP-galactose were incubated with crude enzyme from the taproots of ‘Kuroda’ (A) or ‘Deep purple’ (B); pelargonidin and UDP-galactose were incubated with crude enzyme from the taproots of ‘Kuroda’(C) or ‘Deep purple’(D).


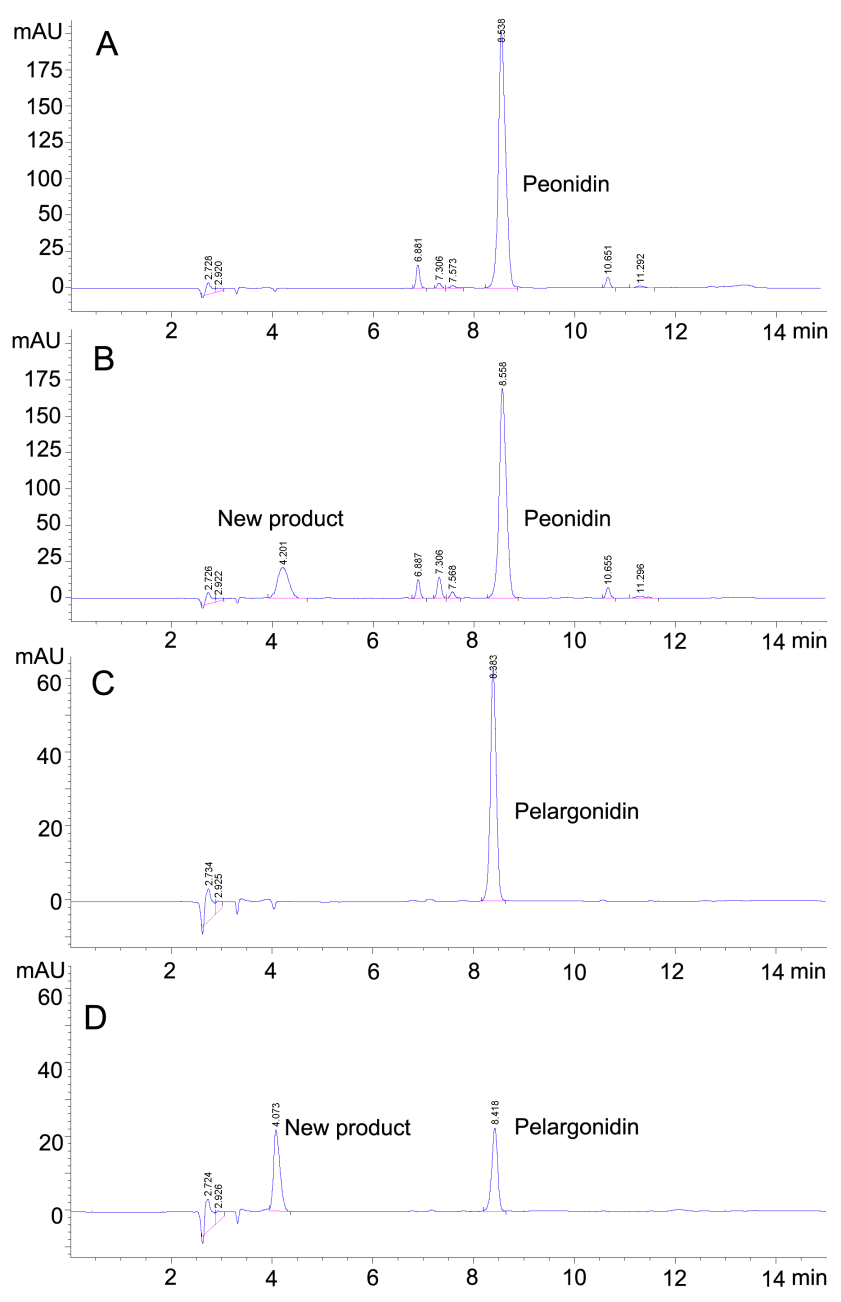

Supplement: Supplementary Information [file srep27356-s1.doc]
